# Robustness in an Ultrasensitive Motor

**DOI:** 10.1128/mBio.03050-19

**Published:** 2020-03-03

**Authors:** Guangzhe Liu, Antai Tao, Rongjing Zhang, Junhua Yuan

**Affiliations:** a Hefei National Laboratory for Physical Sciences at the Microscale and Department of Physics, University of Science and Technology of China, Hefei, Anhui, China; University of Utah; Massachusetts Institute of Technology

**Keywords:** sensitivity, molecular motor, adaptive remodeling, flagellar motor

## Abstract

In Escherichia coli, the chemotaxis response regulator CheY-P binds to FliM, a component of the switch complex at the base of the bacterial flagellar motor, to modulate the direction of motor rotation. The bacterial flagellar motor is ultrasensitive to the concentration of unbound CheY-P in the cytoplasm. CheY-P binds to FliM molecules both in the cytoplasm and on the motor. As the concentration of FliM unavoidably varies from cell to cell, leading to a variation of unbound CheY-P concentration in the cytoplasm, this raises the question whether the flagellar motor is robust against this variation, that is, whether the rotational bias of the motor is more or less constant as the concentration of FliM varies. Here, we showed that the motor is robust against variations of the concentration of FliM. We identified adaptive remodeling of the motor as the mechanism for this robustness. As the level of FliM molecules changes, resulting in different amounts of the unbound CheY-P molecules, the motor adaptively changes the composition of its switch complex to compensate for this effect.

## Abstract (precis)

The bacterial flagellar motor is an ultrasensitive motor. Its output, the probability of the motor turning clockwise, depends sensitively on the occupancy of the protein FliM (a component on the switch complex of the motor) by the input CheY-P molecules. With a limited cellular pool of CheY-P molecules, cell-to-cell variation of the FliM level would lead to large unwanted variation of the motor output if not compensated. Here, we showed that the motor output is robust against the variation of FliM level and identified the adaptive remodeling of the motor switch complex as the mechanism for this robustness.

## INTRODUCTION

Many protein complexes in biology are highly sensitive to the concentration of their ligands (1–5). The ligand binds to a substrate molecule on the complex to generate a sensitive response. This sensitivity is usually described as a highly sensitive dependence of the response on the concentration of unbound ligands. The concentration of the substrates unavoidably varies from cell to cell, leading to a variation of the concentration of unbound ligands, thereby raising the question whether the response of the complex is robust against this variation of substrate concentration.

This issue of robustness can also be represented in the following way. The sensitivity of the complex can also be described as a sensitive dependence of the response on the occupancy of the substrates (defined as the probability of binding a ligand for each substrate). With a limited pool of ligands, as the concentration of the substrates changes from cell to cell, this occupancy would change, thereby changing the output dramatically due to the high sensitivity.

Here, we investigated this problem in the bacterial flagellar motor, an ultrasensitive protein complex (5). The flagellar motor is a transmembrane machine that drives the rotation of a long helical filament, propelling the swimming of bacteria (6). The bacterial flagellar motor is the downstream of the bacterial chemotaxis pathway. It is a reversible rotatory motor that stochastically changes its rotational direction between counterclockwise (CCW) and clockwise (CW), thereby changing the swimming modes of the bacteria, which alternate between run and tumble. The response regulator of the chemotaxis pathway, the phosphorylated form of CheY (designated CheY-P), binds to a component of the switch complex at the base of the flagellar motor, FliM, increasing the fraction of time that the motor spins CW (raising the CW bias) (7, 8). The output of the flagellar motor, the CW bias, was found to be ultrasensitive to its input, the concentration of unbound CheY-P, with a Hill coefficient in the relationship of CW bias versus [CheY-P] measured to be as high as 21 (9). This ultrasensitivity plays an important role in signal amplification in bacterial chemotaxis. At the same time, it poses a serious problem for the steady-state output of the motor in the face of cell-to-cell variation of FliM concentration. At a specific total steady-state [CheY-P], cell-to-cell variation of [FliM] would lead to variation in the unbound [CheY-P], resulting in a large cell-to-cell variation in the steady-state CW bias if not compensated.

It was shown previously that the motor dynamically remodels the composition of its switch complex (10–16) and that this adaptive remodeling offers robustness of the motor response against cell-to-cell variation of steady-state CheY-P concentration (10). Here, we found that it also offers robustness against cell-to-cell variation of FliM concentration.

## RESULTS

### Population distribution of CW bias for wild-type cells is drastically different from prediction.

**FIG 1:**
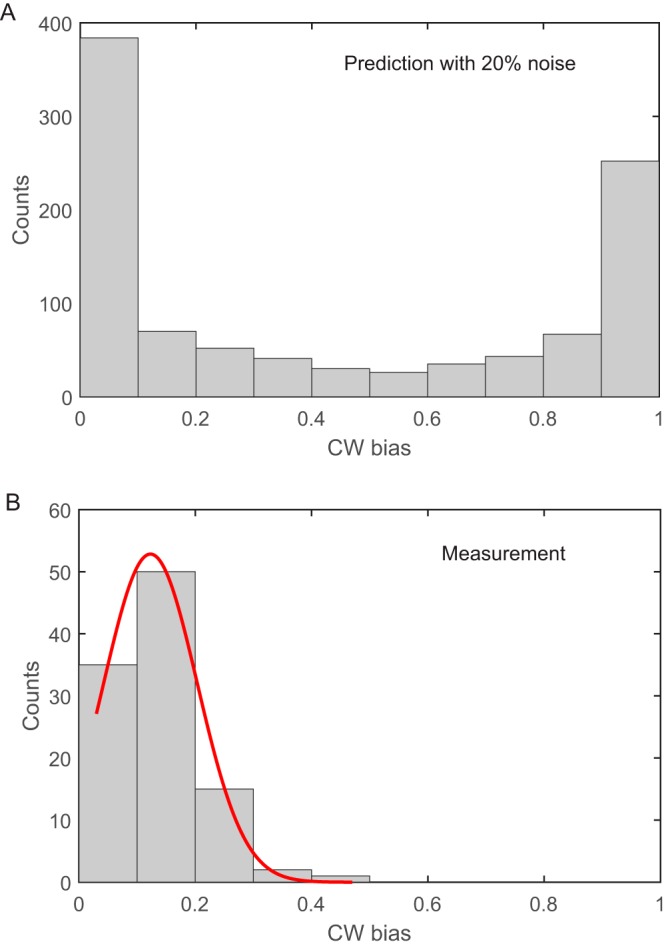
Distributions of CW bias for wild-type cells. (A) The distribution predicted by assuming 20% uncorrelated noises for levels of CheY-P and FliM. (B) Measurements from 103 wild-type cells. The red line is a Gaussian fit.

We sought to investigate the possible effects of [FliM] noise (cell-to-cell variation of [FliM]) on the steady-state motor CW bias. At steady state, there are cell-to-cell variations of [CheY-P] due to noises in gene expression and chemotaxis signaling and of [FliM] due to noises in gene expression. Some of the cell-to-cell variations in [CheY-P] and [FliM] are uncorrelated as *cheY* and *fliM* are on different operons and there are additional contributions to [CheY-P] variations from chemotaxis signaling. To estimate the effect of these variations on the population distribution of CW bias in wild-type Escherichia coli K-12 cells, we used a conservative estimate of the intrinsic (uncorrelated) noise for both [CheY-P] and [FliM] of 20% (17–19) and with average levels of CheY-P and FliM equal to 4.1 and 2.0 μM, respectively (8, 20). After subtracting the fraction of CheY-P bound to FliM (both cytoplasmic and in-motor), we obtained the level of unbound CheY-P molecules in each cell. Using the motor response curve (CW bias versus [CheY-P]) for adapted motors measured by Cluzel and coworkers (5), we extracted the predicted population distribution of CW bias (Fig. 1A). Most cells would exhibit CW bias of 0 or 1, unable to maintain their chemotactic sensitivity. We therefore suspected that there must be mechanisms which offer robustness against these variations. In fact, we experimentally measured the population distribution of CW bias in wild-type cells and found that it is dramatically different from the prediction, with a peak around 0.12 (Fig. 1B). As motor adaptation was discovered to offer robustness against cell-to-cell variation of [CheY-P] (10), and we have already included this robustness by using the motor response curve for adapted motor (with a Hill coefficient of 10.3) in the prediction, Fig. 1A shows mostly the effect of variation in [FliM] if there was no mechanism of robustness against this variation. The dramatic difference between Fig. 1A and B testified to the existence of mechanisms for this robustness, which we sought to understand in this study.

### Population distributions of CW bias for cells with different FliM expression levels are similar.

**FIG 2:**
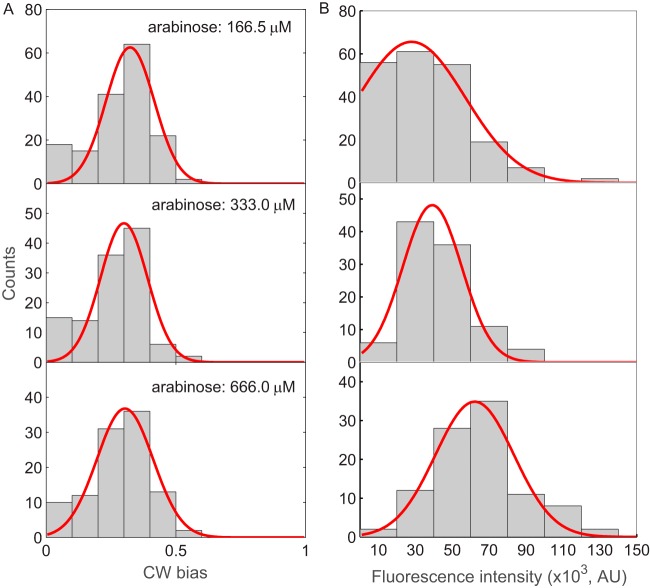
(A) CW bias distributions for Δ*fliM* cells with FliM-eGFP induced with different amounts of the inducer arabinose. The numbers of cells measured were 162, 118, and 104 for the arabinose levels of 166.5, 333.0, and 666.0 μM, respectively. (B) Distributions of total fluorescence intensities in the FCS volume (proportional to the total levels of FliM-eGFP in individual cells) at different levels of the inducer arabinose. The numbers of cells measured were 200, 100, and 100 for the arabinose levels of 166.5, 333.0, and 666.0 μM, respectively. The red lines are Gaussian fits.

To further demonstrate this robustness against variation in FliM levels, we expressed FliM-enhanced green fluorescent protein (eGFP) from a medium-copy-number plasmid under the control of the pBAD promoter, in a Δ*fliM* E. coli K-12 strain. By adjusting the concentration of the inducer (arabinose), we obtained three populations of cells with different average levels of FliM expression. The motors with FliM-eGFP fusion behave normally with similar rotational speed as the wild-type motors, and cells expressing wild-type FliM or FliM-eGFP behave similarly on a swim plate (see Fig. S1A in the supplemental material). We measured the distributions of motor CW bias for the three populations of cells, finding that they are very similar as shown in Fig. 2A, with fitted Gaussian functions at 0.33 ± 0.10, 0.30 ± 0.09, and 0.31 ± 0.12 (peak ± standard deviation [SD]) for inducer levels of 166.5, 333.0, and 666.0 μM arabinose, respectively. Motors with FliM-eGFP showed larger average CW bias than motors with wild-type FliM.

10.1128/mBio.03050-19.2 FIG S1 (A) Similar behavior of cells of HCB1645 (Δ*fliM*) carrying pBAD33FliM (left) or pBAD33FliM-eGFP (right) on a TB swim plate (0.27% agar) after incubation at 30°C for 8 h. (B) Schematic of the FCS setup. Download FIG S1, TIF file, 0.3 MB. Copyright © 2020 Liu et al. 2020 Liu et al. This content is distributed under the terms of the Creative Commons Attribution 4.0 International license.

Considering the fact that CheY-P binds to FliN in addition to FliM (21) and that FliM forms a complex with FliN in the cytoplasm (13), we also tried to test whether different levels of coexpression of FliM and FliN affect the CW bias of the flagellar motor. To adjust the concentration of FliM-eGFP and FliN simultaneously, we constructed the plasmid pBAD33FliM-eGFP&FliN and transformed it into GL4 (Δ*fliC fliM fliN*). We obtained three populations of cells with different average levels of FliM-eGFP and FliN expression by adjusting the concentration of the inducer arabinose. We experimentally measured the distributions of motor CW bias for the three populations of cells, finding that they are very similar as shown in Fig. S2 with fitted Gaussian functions at 0.22 ± 0.12, 0.20 ± 0.14, and 0.21 ± 0.12 (peak ± SD) for inducer levels of 166.5, 333.0, and 666.0 μM arabinose, respectively. The results show that the switching properties of the flagellar motor do not change with different levels of coexpression of FliM and FliN. The difference between the peak CW bias for the two strains (Δ*fliM* mutant expressing FliM-eGFP and Δ*fliM fliN* mutant expressing FliM-eGFP and FliN) might be due to strain differences.

10.1128/mBio.03050-19.3 FIG S2 CW bias distributions for Δ*fliM fliN* cells with FliM-eGFP&FliN induced with different amounts of the inducer arabinose. Download FIG S2, EPS file, 0.3 MB. Copyright © 2020 Liu et al. 2020 Liu et al. This content is distributed under the terms of the Creative Commons Attribution 4.0 International license.

### Motor CW bias is independent of cytoplasmic FliM concentration.

We built a fluorescence correlation spectroscopy (FCS) setup to monitor the cellular FliM level (22–25). Details of the setup are presented in Materials and Methods, and a schematic of the setup is shown in Fig. S1B. We focused the excitation laser beam to a diffraction-limited spot on the cell and collected the eGFP fluorescence emissions in a confocal geometry with an avalanche photodiode. The confocal volume is about 1.5 μm in axial half-length, which is larger than the cell thickness. So, on average, the fluorescent intensity in the confocal volume includes emissions from a fraction of both the free FliM-eGFP molecules and FliM-eGFP molecules assembled in motors in the membrane, that is, it is proportional to the total FliM level. The absolute concentration of free FliM molecules can be extracted from fluctuations of the fluorescence intensity by calculating an autocorrelation function. We calibrated the focal volume of the FCS setup using a solution of the dye molecule Alexa 488 with a known diffusion coefficient (Fig. S3A). By fitting the autocorrelation function of the FCS signal for Alexa 488 with a theoretical function describing translational diffusion in a three-dimensional Gaussian volume, we extracted the lateral radius of the focal volume to be 0.3 μm in our setup. A typical autocorrelation function measured for diffusing FliM-eGFP molecules in a single cell is shown in Fig. S3B. By fitting the autocorrelation function with a theoretical function describing two-dimensional translational diffusion, we extracted the diffusion coefficient and the number of freely diffusive FliM-eGFP molecules inside the focal volume (with the axial length limited by the cell thickness). We noted that the level of freely diffusive FliM molecules that we extracted included freely diffusing cytoplasmic FliM molecules both bound and unbound with CheY-P.

10.1128/mBio.03050-19.4 FIG S3 (A) Typical autocorrelation function measured for Alexa 488 molecules diffusing in water. The red line is a fit with a theoretical function describing three-dimensional translational diffusion. (B and C) Typical autocorrelation function measured for diffusing FliM-eGFP in a single cell. The red line is a fit with a theoretical function describing two-dimensional translational diffusion (B) or restricted 2-D diffusion (C). Download FIG S3, EPS file, 0.3 MB. Copyright © 2020 Liu et al. 2020 Liu et al. This content is distributed under the terms of the Creative Commons Attribution 4.0 International license.

We plotted the distributions of fluorescence intensities for the three populations of cells induced with 166.5, 333.0, and 666.0 μM arabinose, finding that the distributions shifted right as the induction level increased (Fig. 2B). The mean intensities are (3.59 ± 0.17) × 10^4^, (4.17 ± 0.17) × 10^4^, and (6.72 ± 0.28) × 10^4^ photons/s (mean ± standard error of the mean [SEM]) for inducer levels of 166.5, 333.0, and 666.0 μM arabinose, respectively. Therefore, as expected, the total concentration of FliM molecules increases with higher induction levels. These levels of expression were about 80 to 160% of the level of the native FliM molecules in a wild-type cell (26).

**FIG 3:**
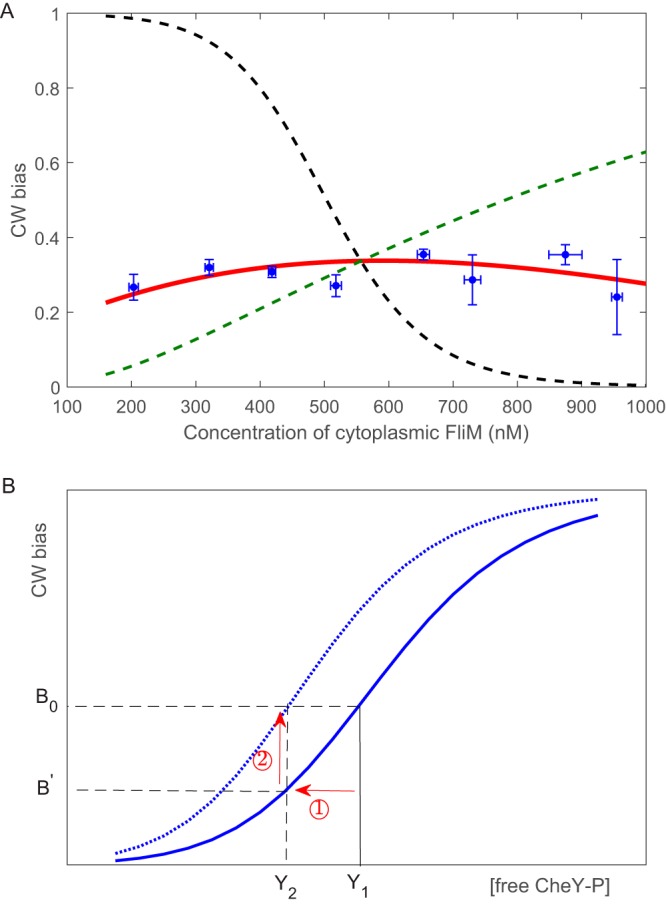
(A) The CW bias as a function of the cytoplasmic levels of FliM extracted by simultaneous measurements of motor CW bias and cytoplasmic FliM concentration on individual cells (103 cells, blue circles with error bars). The black dashed line is the calculation with the sole effect of CheY-P binding to FliM (see ① in panel B), the green dashed line is the calculation with the sole effect of motor remodeling (see ② in panel B), and the red line is the calculation combining the two effects. (B) Schematic of the mechanism of robustness. At a specific total steady-state CheY-P concentration, when FliM level increases, the unbound CheY-P level decreases (①), and motor remodeling includes more FliM molecules in the C ring, shifting the curve left (dotted curve) and thus increasing the CW bias (②).

To directly monitor the effect of cell-to-cell variation of freely diffusive FliM level on the motor output, we measured both the concentration of freely diffusive FliM molecules using FCS and the motor CW bias using a bead assay, for individual cells induced with 166.5 μM arabinose. We measured for 103 cells, which covered a wide range of freely diffusive FliM concentrations. We sorted the free FliM concentrations into eight groups and calculated the average CW bias and FliM concentration within each group. We plotted the CW bias as a function of the concentration of freely diffusive FliM molecules (filled circles with error bars in Fig. 3A), finding that the CW bias is nearly independent of the FliM concentration. This directly demonstrated the robustness of the motor output against variation in FliM concentration.

### Understanding robustness with a model of motor adaptive remodeling.

We sought to understand the mechanism of this robustness. As outlined in Fig. 3B in the plot of CW bias versus unbound CheY-P concentration, with a fixed total [CheY-P], the unbound [CheY-P] would reduce from *Y*_1_ to *Y*_2_ when the total FliM concentration increased (denoted by ①), and the CW bias would reduce from *B*_0_ to *B*′. However, motor adaptive remodeling would compensate for this reduction by shifting the motor response curve to the left (dotted curve in Fig. 3B), thereby increasing the CW bias (denoted by ②). This compensation due to motor remodeling actually included two parts: first, there would be more FliM molecules included in the switch complex to partially adapt for the reduction of CW bias, even if the total FliM concentration did not change; second, as the total FliM concentration did increase (and so did the cytoplasmic FliM concentration [see Fig. S4]), more FliM molecules would be included in the switch complex due to balance of the FliM on/off rates (the law of mass action). We calculated the CW bias as a function of cytoplasmic FliM concentration using the model of motor remodeling (27). Details of the model and calculations are presented in Materials and Methods. By calculating the sole effect of CheY-P binding to FliM, the CW bias reduces as the cytoplasmic FliM concentration increases (black dashed line in Fig. 3A), whereas it increases with the cytoplasmic FliM concentration if calculating the sole effect of motor remodeling (green dashed line in Fig. 3A). By combining the two effects, we obtained the near-independence of the CW bias on the cytoplasmic FliM concentration (red solid line in Fig. 3A).

10.1128/mBio.03050-19.5 FIG S4 Concentration of total FliM as a function of the concentration of cytoplasmic FliM from the FCS measurements. The blue dots are experimental data, and the red line is a linear fit resulting in [FliM]_total_ = 5.635 × [FliM]_free_ + 503.9. The concentration of total FliM was converted from the fluorescence intensity by using the calibration that the mean total FliM level is 2,000 nM when induced with 166.5 μM arabinose. Download FIG S4, EPS file, 0.2 MB. Copyright © 2020 Liu et al. 2020 Liu et al. This content is distributed under the terms of the Creative Commons Attribution 4.0 International license.

### Directly testing the mechanisms in the model of motor adaptive remodeling.

We sought to directly demonstrate the existence of the two competing effects, namely, that the number of FliM molecules assembled in the motor increases as the cytoplasmic FliM concentration increases (motor remodeling) and that CheY-P binds to free cytosolic FliM (unbound [CheY-P] changes with [FliM]). For the first effect, we would express FliM-eGFP at specific induction levels and correlate the motor fluorescence with cytoplasmic fluorescence for individual cells. For the second effect, we would compare the CheY-P diffusion coefficients with and without FliM expression. To avoid possible effect of FliM aggregates, seen previously at cell poles with high expression of FliM (26), we expressed FliM-eGFP at a moderate induction level (with 166.5 μM arabinose) and compared the fluorescence images of cells with and without assembled motors (Δ*fliG*). As shown in Fig. S5, there are no FliM aggregates in cells without assembled motors at this expression level, and the motor spots are clearly seen in cells with assembled motors. We used this expression level of FliM for the following experiments.

10.1128/mBio.03050-19.6 FIG S5 Fluorescence images of cells expressing FliM-eGFP from the plasmid pBAD33FliM-eGFP induced with 166.5 μM arabinose. (A) Epifluorescence image of cells (Δ*fliG fliM*) without assembled motors. (B) TIRF image of cells (Δ*fliG fliM*) without assembled motors. (C) TIRF images of cell (Δ*fliM*) with assembled motors. Download FIG S5, TIF file, 0.5 MB. Copyright © 2020 Liu et al. 2020 Liu et al. This content is distributed under the terms of the Creative Commons Attribution 4.0 International license.

**FIG 4:**
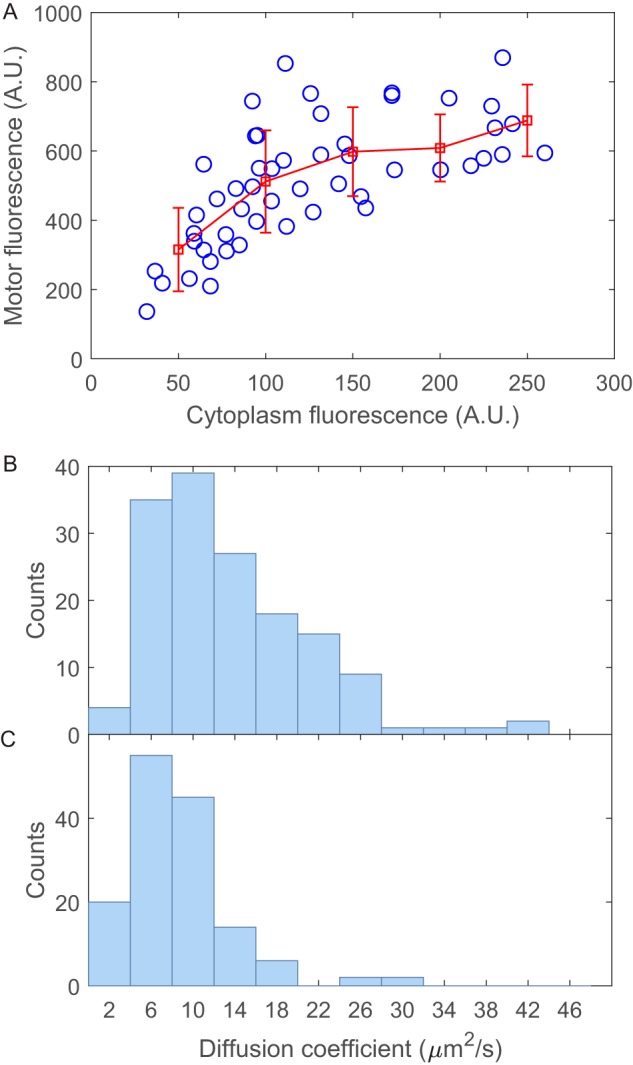
(A) Motor fluorescence intensity increases as the cytoplasmic fluorescence intensity increases, as observed in a TIRF setup for Δ*fliM* cells with FliM-eGFP expressed from plasmid. The red symbols are the mean and SD after binning the data into bin sizes of 50. (B and C) Distributions of CheY-P diffusion coefficients in cytoplasm for different expression levels of FliM which were induced with 0 and 166.5 μM arabinose for panels B and C, respectively. The numbers of cells measured are 152 and 144 for panels B and C, respectively.

We first tested whether the motor fluorescent spot gets brighter with increasing cytoplasmic FliM concentration. We expressed FliM-eGFP in a CCW-rotating Δ*fliM* strain and made sure that the motor rotated 100% CCW by using a tethered-cell assay. We then monitored individual tethered cells for both motor fluorescence with total internal-reflection fluorescence (TIRF) microscopy and cytoplasmic fluorescence with epifluorescence microscopy. The population of cells would exhibit a wide range of cytoplasmic fluorescence intensities. We counted only motors identified as the centers of rotation of tethered cells. The motor fluorescence as a function of the cytoplasmic fluorescence measured from 51 cells is shown in Fig. 4A, clearly showing that the number of FliM molecules in a motor increases as the cytoplasmic FliM concentration increases. We can fit the data with the law of mass action to extract the ratio of FliM off and on rates, *k*_off_/*k*_on_ (Fig. S7), resulting in a ratio of 914 ± 178 nM. As the cytoplasmic FliM concentration (*U*) in a wild-type strain is about several hundred nanomolar, *k*_off_ is at the same level of *k*_on_*U* for a wild-type cell. This is consistent with previous estimates (10, 12). Therefore, we directly demonstrated that the motor remodels in response to changes in FliM concentration: as FliM level increases, more FliM molecules are assembled in the motor.

As the FliM-FliN complex likely behaved as an exchange unit for adaptive remodeling (13), we also repeated the above measurements by coexpressing FliM-eGFP and FliN in a CCW-rotating Δ*fliM FliN* strain. The experiments showed similar increase of the number of FliM molecules in the motor as the FliM-eGFP and FliN levels increased, with a similar value of the ratio of *k*_off_/*k*_on_ (936 ± 196 nM) as above (Fig. S8). This suggested that in the range of FliM levels we tested above, the wild-type level of FliN may be sufficient for forming complexes with FliM.

We then tested whether CheY-P binds to free cytosolic FliM. We used a Δ*cheRB cheZ cheY fliM fliG* strain (in which essentially all CheY molecules were phosphorylated) and expressed wild-type FliM from a plasmid under the control of the arabinose-inducible pBAD promoter and CheY-eGFP from a plasmid under the control of the isopropyl-β-d-thiogalactoside (IPTG)-inducible pTrc promoter (induced with 0.02 mM IPTG). The deletion of *fliG* was to make sure there were no assembled motors in cells (Fig. S5A), so that CheY-P bound only to cytosolic FliM. We compared two cases: without arabinose induction and with 166.5 μM arabinose induction of FliM. We measured the CheY-eGFP diffusion coefficients in the cytoplasm under the two situations using the FCS setup. The distributions of CheY-eGFP diffusion coefficients are shown in Fig. 4B and C. The means ± SEM are 13.5 ± 0.6 and 8.7 ± 0.4 μm^2^/s for no induction and with 166.5 μM arabinose induction, respectively. Therefore, expression of FliM molecules decreases the CheY-eGFP diffusion coefficients in the cytoplasm, through binding of the two molecules.

## DISCUSSION

In summary, we found that the motor output (CW bias) is surprisingly robust against changes in the concentration of FliM molecules, the binding partner of the motor input (CheY-P). As the total level of expression for FliM increased by 2-fold, the population distribution of motor CW bias remained unchanged. With cell-to-cell variation of cytoplasmic FliM concentration, the CW bias remained nearly constant as the cytoplasmic FliM concentration changed by 5-fold. We discovered that motor adaptive remodeling is the mechanism for this robustness. We also directly demonstrated the existence of the two competing effects for this robustness: CheY-P binds to cytosolic FliM molecules and the motor remodels in response to changes in FliM level.

A possible mechanism that could also contribute to this robustness was that the binding affinity of the cytoplasmic FliM-FliN for CheY-P might be quite low compared to that of the C ring. However, the binding curve for CheY-P to FliM-FliN was measured previously where a large fraction of the FliM-FliN complexes were in the cytoplasm (8), leading to a dissociation constant similar to the *K*_1/2_ of the motor CW bias versus [CheY-P] Hill curve measured in another study (5). *K*_1/2_ is the average of the dissociation constants for CheY-P binding to the C ring in CW and CCW states. Therefore, these studies showed that the binding affinities of CheY-P for cytoplasmic FliM-FliN and for the C ring are comparable.

Another mechanism was proposed previously, in which the unbound [CheY-P] variation was suppressed when [FliM] varies, due to the fact that CheZ dephosphorylates only free CheY-P molecules, and thus CheY-P binding to FliM increases the CheY-P lifetime (28). We further included this mechanism in our model (details in Text S1 in the supplemental material) and found that it alone cannot explain the robustness observed here but, when combined with the mechanism of motor adaptive remodeling, can fully explain the robustness of motor response against changes in FliM concentration (Fig. S9).

CheY-P binds to both FliM and FliN in the FliM-FliN complexes (21). For simplicity, our model has been described as binding of CheY-P to FliM only. Nevertheless, the effect of CheY-P also binding to FliN was implicitly included, by using 3.1 μM as the value of the dissociation constant of CheY-P binding to FliM. This value was determined by previous experiments as the dissociation constant of CheY-P binding to the FliM-FliN complex and also as the *K*_1/2_ value in the CW bias-versus-[CheY-P] Hill curve (5, 8).

For a highly sensitive molecular machine, variation of concentration of the substrates changes the level of unbound ligands, thereby changing the response of the machine drastically. Cell-to-cell variation of protein concentration is ubiquitous in biology. Variation of concentration of the substrates will lead to huge unwanted cell-to-cell variation of the machine response if not compensated. Here, we discovered that motor adaptive remodeling in the bacterial flagellar motor serves as a mechanism to compensate this effect, leading to robustness of the motor output against cell-to-cell variation of the concentration of the substrate. Similar mechanisms of robustness by adaptive remodeling should exist in other sensitive protein complexes and signal transduction pathways.

## MATERIALS AND METHODS

### Strains and plasmids.

Strains GL1 (Δ*fliC fliM*), GL2 (Δ*fliC fliM cheY*), JY26 (Δ*fliC*), GL3 (Δ*fliM* Δ*fliG* Δ*tap*-*cheZ*), GL4 (Δ*fliC fliM fliN*), and GL5 (Δ*fliC fliM fliN cheY*) are derivatives of E. coli K-12 strain RP437. A C-terminal fusion of eGFP (with the A206K mutation to eliminate self-association [29]) to FliM was constructed using a short amino acid linker (5× glycine), and the fusion gene was cloned into the pBAD33 vector under the control of an arabinose-inducible promoter (30), yielding the plasmid pBAD33FliM-eGFP. The wild-type *fliN* gene was cloned into pBAD33FliM-eGFP to make the plasmid pBAD33FliM-eGFP&FliN, which expresses FliM-eGFP and FliN from an arabinose-inducible promoter in the same order as they are positioned on the chromosome and with their native ribosome-binding sequences. The wild-type *fliM* gene was cloned into the pBAD33 vector to make the plasmid pBAD33FliM. A C-terminal fusion of eGFP to CheY was constructed using a 5× glycine linker, and the fusion gene was cloned into the pTrc99a vector under the control of an IPTG-inducible promoter, yielding the plasmid pTrc99aCheY-eGFP. The plasmid pKAF131 constitutively expresses the sticky filament FliC^st^. The plasmid pFD313 also constitutively expresses FliC^st^ and is compatible with pBAD33FliM-eGFP (31). JY26 carrying pKAF131 was used to measure population distribution of CW bias for wild-type cells. For FCS measurements and CW bias measurements with adjustable expression levels of FliM, GL1 carrying the plasmids pFD313 and pBAD33FliM-eGFP was used. GL4 carrying the plasmids pBAD33FliM-eGFP&FliN and pFD313 was also used for CW bias measurements. For TIRF measurements, GL2 carrying the plasmid pBAD33FliM-eGFP and GL5 carrying the plasmid pBAD33FliM-eGFP&FliN were used. For measuring CheY-P diffusion coefficients in cytoplasm with and without expression of FliM, GL3 carrying the plasmids pBAD33FliM and pTrc99aCheY-eGFP was used.

### Cell culture.

Cells were grown at 30°C in T broth (1% tryptone and 0.5% NaCl) with the appropriate antibiotics (100 μg/ml ampicillin, 25 μg/ml chloramphenicol) and various amounts of the inducer arabinose to an optical density at 600-nm wavelength of about 0.45, washed three times with motility buffer (10 mM potassium phosphate, 0.1 mM EDTA, 1 mM methionine, 10 mM lactate, pH 7.0), sheared to truncate flagella, and concentrated by a factor of 5. They were used immediately for experiments or stored at 4°C for up to 2 h. All experiments were carried out at 23°C.

### CW bias measurements.

Motor CW biases were measured using a bead assay. Sheared cells were immobilized on a glass coverslip coated with poly-l-lysine (0.01%, P4707; Sigma, St. Louis, MO), and diluted 1.0-μm-diameter polystyrene latex beads (2.69%, 07310; Polysciences, Warrington, PA) were attached to the truncated flagella. The polystyrene beads were observed by phase-contrast microscopy using a Nikon Ti-E inverted microscope. The motion of the beads was recorded with a complementary metal oxide semiconductor (CMOS) camera (DCC1545M-GL; Thorlabs, Newton, NJ) at 500 frames per second with a reduced region of interest that covered selected beads. Data analysis was done by using custom scripts in Matlab. Using a threshold-crossing algorithm (32), the velocity time series were converted to binary time series indicating the rotational directions as a function of time, and the CW bias was computed as the ratio of the time spent in CW to the total time duration.

### FCS setup.

A schematic diagram of the FCS setup is shown in Fig. S1B in the supplemental material. Laser light was introduced into a Nikon Ti-E inverted microscope through the rear port, and fluorescence emissions were detected through the camera port. Light from a 488-nm laser (Sapphire488LP; Coherent) was attenuated to 20 μW for FCS in live cell (50 μW for calibration and 10 μW for fluorescence intensity measurement in live cell) to minimize bleaching of the dye, expanded into a parallel beam about 7 mm in diameter, reflected by a dichroic mirror (DM505; Chroma), and focused to a diffraction-limited spot with an oil-immersion lens objective (Nikon TIRF objective, 100×; numerical aperture [NA], 1.49). The fluorescence emissions from the sample were collected by the same objective, transmitted through the dichroic mirror and an emission filter (BA510-560; Chroma), and focused by a lens onto the core of a multimode optical fiber (core diameter, 50 μm). The optical fiber was connected to an avalanche photodiode (SPCM-AQRH-24-FC; Excelitas Technologies) for single photon detection, which generated a transistor-transistor logic (TTL) pulse for each detected photon. The signal was then processed in real time by a multiple-tau hardware correlator (Flex02-01D; Correlator.com) that generates an autocorrelation function with quasilogarithmic lag times. The light for bright-field microscopy was provided by a tungsten lamp illuminating the sample from above, using a colored glass filter (FGL610S; Thorlabs) to allow passage of light with a wavelength larger than 610 nm.

### FCS measurements and analysis.

To ensure that the FCS focal volume always centered along the thickness of the cell body, we tested the fluorescent intensities by moving the axial position of the objective in steps of 0.25 μm and recorded the photon counts in 1 s. The position with the highest photon count was determined as the center position along the thickness of the cell body. The fluorescence signal at the center position in a single FCS measurement was then recorded for 10 s, and the autocorrelation function for the signal was generated by the hardware correlator. We first calibrated the focal volume with a solution of the dye molecule Alexa 488 with a known diffusion coefficient (400 μm^2^/s). The FCS signal for the Alexa 488 solution was recorded, and the theoretical autocorrelation function describing translational diffusion in a three-dimensional Gaussian volume (33)

$$G(\tau )=\frac{1}{N}\cdot \left[\frac{1}{1+\tau /\tau_{D}}\cdot \frac{1}{\sqrt{1+(r_{0}^{2}/z_{0}^{2})(\tau /\tau_{D})}}\right]$$

was fitted to the measured autocorrelation function (Fig. S3A), where *r*_0_ is the lateral radius and *z*_0_ is axial half-length of the three-dimensional Gaussian volume, and τ*_D_* = *r*_0_^2^/4*D* where *D* is diffusion coefficient of the diffusing molecule. τ*_D_* and *r*_0_/*z*_0_ were extracted from the fitting, and as *D* was known for Alexa 488 molecules, *r*_0_ and *z*_0_ were obtained, resulting in values of 0.3 μm and 1.5 μm for *r*_0_ and *z*_0_, respectively, in our setup. The theoretical autocorrelation function describing two-dimensional translational diffusion

$$G\left(\tau \right)=\frac{1}{N}{\left[1+\frac{4D\tau }{r_{0}^{2}}\right]}^{-1}$$

was fitted to the measured autocorrelation function for FliM-eGFP diffusing in cells, where 2*r*_0_ = 0.6 μm is the diameter of the detection volume, *D* is the cytoplasmic diffusion coefficient for FliM-eGFP, and *N* is the number of molecules inside the detection volume. One molecule in this volume represented a concentration of 7 nM using a cell thickness of 0.8 μm. The values of *D* and *N* (which was converted to concentration of freely diffusive molecules) were obtained from the fitting. A typical autocorrelation function along with the fit for FliM-eGFP diffusing inside a cell is shown in Fig. S3B. The average diffusion constant of the cytoplasmic FliM-eGFP fusion was found to be 9.6 ± 3.2 μm^2^/s (mean ± SD) from measurements in 299 cells. We also tried fitting the measured autocorrelation functions with a theoretical autocorrelation function

$$G\left(\text{τ}\right)=\frac{1}{N}{\left[1+{\left(\frac{\text{τ}}{{\text{τ}}_{a}}\right)}^{\text{α}}\right]}^{-1}$$

which describes restricted two-dimensional translational diffusion (<*r*^2^> = 4Γ*t*^α^) in the cytoplasm, where τ*_a_^α^* = *r*_0_^2^/4Γ, Γ is the anomalous transport coefficient, and α is the anomalous exponent characterizing restricted diffusion (34). The fitted *N*’s are similar whether using restricted diffusion or not. The average fitted Γ and α were found to be 2.7 ± 2.2 μm^2^/s^α^ and 0.69 ± 0.11 (mean ± SD), respectively. Data fitting was performed with Matlab, by minimizing the weighted reduced χ^2^ value using the Levenberg-Marquardt nonlinear least-squares algorithm.

### Predicting the distribution of wild-type CW bias.

The expression levels of FliM and CheY-P in individual cells were generated assuming a summation of the extrinsic (correlated) and intrinsic (uncorrelated) noises. Therefore, the following equations were used (17):

$$x_{i}=<\text{x\_i}>{}_{\text{wt}}\left(r_{\text{ex}}+\nu {\text{ξ}}_{i}^{\left(2\right)}\right),\;$$

$$r_{\text{ex}}=N_{r}\text{exp}\left[{\text{αξ}}^{\left(1\right)}ln10\right]\;$$

where *r*_ex_ is the extrinsic noise with α = 0.2 and *N_r_* was chosen such that <*r*_ex_> = 1, ξ^(1)^ and ξ^(2)^ are normally distributed random variables with zero mean and variance of 1, ν = 0.2 specifies a level of 20% for the intrinsic noise (17–19), *x_i_* is the concentration of FliM or CheY-P, and <*x_i_*>_wt_ is the corresponding average concentration of *x_i_*. The average concentrations of FliM and CheY-P were chosen to be 2,000 nM and 4,100 nM, respectively (8, 20). Although the total cellular CheY concentration may be higher (19), the phosphorylated fraction should be in this range (from additional evidence of the motor CW bias-versus-CheY-P concentration curve). For each cell at specific values of *Y*_tot_ (total concentration of CheY-P) and [FliM] (total concentration of FliM), the concentration of unbound CheY-P (*Y*_ub_) was calculated by solving the equation

(1) $$Y_{\mathrm{ub}}=Y_{\mathrm{tot}}-[\mathrm{FliM}]\times \frac{{Y_{\mathrm{ub}}}^{n}}{\left({Y_{\mathrm{ub}}}^{n}+{K_{\frac{1}{2}}}^{n}\right)}$$

where *n *= 1.7 was the Hill coefficient and *K*_1/2_ = 3,100 nM was the dissociation constant for binding of CheY-P and FliM measured previously (8). The CW bias was then extracted from the motor response curve measured by Cluzel et al. using the value of *Y*_ub_ (5). The predicted CW bias distribution for the wild-type cells is shown in Fig. 1A, with simulations performed for 1,000 individual cells.

### Stochastic simulation of motor remodeling.

According to the model of motor adaptive remodeling (12, 27), the time rate of change of the number of FliM molecules (*N*) in a motor is determined by the balance of FliM molecules coming on and off the motor:

(2) $$\frac{\mathrm{dN}}{\mathrm{dt}}=k_{\text{on}}U\left(M-N\right)-k_{\text{off}}\left(N-N_{\text{NE}}\right)$$

where *M* is the maximum number of binding sites for the FliM molecules in the motor switch complex, *U* is the concentration of cytoplasmic FliM molecules unassembled to the motor, *k*_on_ and *k*_off_ are the on and off rates, respectively, and *N*_NE_ is the number of FliM molecules in the motor that do not exchange with the cytoplasmic pool. The value of *N*_NE_ depends on the motor rotational direction. As the CCW and CW intervals (∼1 s) are much shorter than the exchange timescale of the FliM molecules (1/*k*_off_ ∼ 50 s), a quasiequilibrium approximation can be used, and *N*_NE_ can be written as a function of the CW bias (*B*): *N*_NE_ = *B *× 12 + (1 − *B*) × 34. Inserting this equation into equation 2 leads to

(3) $$\frac{\mathrm{dN}}{\mathrm{dt}}=k_{\text{on}}U\left(M-N\right)-k_{\text{off}}\left(N-\left(B\times 12+\left(1-B\right)\times 34\right)\right)$$

At steady state, the rate of change is zero, so

(4) $$k_{\text{on}}U\left(M-N\right)-k_{\text{off}}\left(N-\left(B\times 12+\left(1-B\right)\times 34\right)\right)=0$$

The motor CW bias is ultrasensitive to the concentration of unbound CheY-P (*Y*_ub_), and the dependence can be expressed using the Monod-Wyman-Changeux (MWC) model (35):

(5) $$B=1/(1+\text{exp}(N\times \ln\left(\frac{1+\frac{Y_{\text{ub}}}{K_{2}}}{1+\frac{Y_{\text{ub}}}{K_{1}}}\right)+\text{ε}))$$

where ε is the energy difference for the CW and CCW states when no CheY-P binds to the switch complex, and *K*_1_ and *K*_2_ are the dissociation constants of CheY-P binding to FliM in the CW and CCW states, respectively, satisfying $\sqrt{K_{1}K_{2}=}3.1\;\text{μ}M.$

The parameter *K*_1_ that we used in this study is 1.28 μM according to a previous estimate (27). The parameters *k*_off_ and *k*_on_*U* are both 0.02 s^−1^ according to previous measurements for cells with a wild-type level of cytoplasmic FliM (*U*) (10, 12). To determine the value of *k*_on_, we used a value of *U* for the wild-type of 600 nM according to our estimation. We determined the total CheY-P level (*Y*_tot_) to be 2,855.2 nM using an average CW bias of 0.3 and the motor response curve measured by Cluzel et al. (5). At a specific total level of FliM, the concentration of unbound CheY-P (*Y*_ub_) can be extracted by solving the equation that described the binding of CheY-P to FliM (equation 1). Then, the steady-state CW bias (*B*) can be extracted by solving the combination of equations 4 and 5 if a value of ε was assumed. To obtain a steady-state CW bias of 0.3 at a wild-type [FliM] of 2,000 nM, we determined the value of ε to be 27.8 *k_B_T*.

From our FCS measurements, the total FliM concentration [FliM] scaled linearly with the cytoplasmic FliM concentration *U* (Fig. S4). To calculate the dependence of motor CW bias on the cytoplasmic FliM concentration (red solid line in Fig. 3A), we did the following. At each value of the cytoplasmic FliM concentration (*U*), the total FliM concentration was obtained from the linear dependence in Fig. S4, the concentration of unbound CheY-P (*Y*_ub_) was calculated by solving equation 1, and then the steady-state CW bias (*B*) was calculated by solving the combination of equations 4 and 5. We also calculated the dependence for the sole effect of CheY-P binding to FliM (① in Fig. 3B), by solving equation 1 to obtain *Y*_ub_ and using a motor response curve with a Hill coefficient of 20.7 as measured previously (9). This led to the black dashed line in Fig. 3A. We calculated the dependence for the sole effect of motor remodeling (② in Fig. 3B), by substituting *Y*_tot_ for *Y*_ub_ in equation 5, and then solving the combination of equations 4 and 5, and this resulted in the green dashed line in Fig. 3A (*Y*_tot_ was adjusted so that the green line passes the intersection of the black and red lines). We tried other values of *U* for the wild-type (300 and 900 nM) to determine the value of *k*_on_, and the calculated dependence of CW bias on the FliM concentration did not change much (Fig. S6).

10.1128/mBio.03050-19.7 FIG S6 The calculated CW bias as a function of cytoplasmic FliM concentration assuming different values of the cytoplasmic FliM concentration in a wild-type cell (*U*), thus resulting in different values of FliM on-rate *k*_on_. Blue circles with error bars are experimental data, and red lines are the calculations. Download FIG S6, EPS file, 0.1 MB. Copyright © 2020 Liu et al. 2020 Liu et al. This content is distributed under the terms of the Creative Commons Attribution 4.0 International license.

10.1128/mBio.03050-19.1 TEXT S1 Supplemental methods. Download Text S1, DOCX file, 0.02 MB. Copyright © 2020 Liu et al. 2020 Liu et al. This content is distributed under the terms of the Creative Commons Attribution 4.0 International license.

10.1128/mBio.03050-19.8 FIG S7 (A) Number of FliM molecules in a motor as a function of FliM cytoplasmic concentration, converted from Fig. 4A by using a mean number of FliM molecules in a motor (*N*) of 44 and a mean FliM cytoplasmic concentration (*U*) of 451 nM. (B) Fitting of 1/*N* versus 1/*U* to extract *k*_off_/*k*_on_. Download FIG S7, EPS file, 0.1 MB. Copyright © 2020 Liu et al. 2020 Liu et al. This content is distributed under the terms of the Creative Commons Attribution 4.0 International license.

10.1128/mBio.03050-19.9 FIG S8 TIRF measurements with coexpression of FliM-eGFP and FliN. (A) Number of FliM molecules in a motor as a function of FliM cytoplasmic concentration. (B) Fitting of 1/*N* versus 1/*U* to extract *k*_off_/*k*_on_. Download FIG S8, EPS file, 0.03 MB. Copyright © 2020 Liu et al. 2020 Liu et al. This content is distributed under the terms of the Creative Commons Attribution 4.0 International license.

10.1128/mBio.03050-19.10 FIG S9 The CW bias as a function of the cytoplasmic levels of FliM extracted by simultaneous measurements of motor CW bias and cytoplasmic FliM concentration on individual cells (103 cells, blue circles with error bars). The green dashed line is the calculation with the effect of CheY-P binding to FliM and that this binding increases CheY-P phosphorylation lifetime, and the red line is the calculation combining these effects with the effect of motor adaptive remodeling. Download FIG S9, EPS file, 0.02 MB. Copyright © 2020 Liu et al. 2020 Liu et al. This content is distributed under the terms of the Creative Commons Attribution 4.0 International license.

### TIRF measurements.

To obtain more stable rotation of tethered cells, we used a Δ*cheY* strain with motors that rotate only CCW, and we tethered the cells through hook using antihook antibody (the flagellar filament gene is deleted with Δ*fliC*). GL2 cells were washed with motility medium and tethered to a glass coverslip using anti-FlgE antibody following the procedure described previously (10). Rotating cells were identified, and the motor fluorescence was monitored using a Nikon Ti-E TIRF microscope equipped with a back-illuminated, cooled (−70°C), electron-multiplying charge-coupled device (CCD) camera (DU897U-CS0-BV; Andor Technology). The 488-nm laser power at the exit of the objective was 0.23 mW. Each motor was recorded for 200 frames at a frame rate of 57.7 frames per second (fps), then the illumination angle of the 488-nm laser was adjusted to vertical (epifluorescence), and the same cell was recorded for 200 frames at a frame rate of 23.4 fps (for cytosolic FliM-eGFP fluorescence). The motor spots were about 450 nm in width. We counted only motors identified as the centers of rotation of tethered cells. The fluorescence centroid for each motor was calculated using a Gaussian mask method described previously (10), with a motor mask diameter of 450 nm. We also calculated the motor centroid with two-dimensional (2-D) Gaussian fitting, and the two methods yielded comparable results. After the centroid was determined, the background intensity was defined as the mean intensity per pixel within a square region of 580 by 580 nm^2^ centering on the motor centroid but external to the motor mask (450 nm in diameter). The motor fluorescence intensity was then calculated as the sum of all pixel intensities within the motor mask after subtraction of the background intensity from each pixel. The fluorescence intensities from the first 10 frames were averaged to generate the motor intensity. The fluorescence intensities for the cytosolic FliM-eGFP molecules were calculated in the unit of intensity per pixel (160 by 160 nm^2^). To determine the cytoplasmic fluorescence more precisely, we divided each pixel into 3 by 3 subpixels and counted only fluorescence intensities in those subpixels that were within 150 nm of the cell body longitudinal axis, >250 nm away from a motor spot, and >500 nm away from the cell ends. The motor intensities as a function of the cytoplasmic fluorescence are shown in Fig. 4A. By using an estimate of mean number of FliM molecules in a CCW motor of 44 (12), and the mean cytoplasmic FliM-eGFP concentration of 451 nM with 166.5 μM arabinose induction, we could convert Fig. 4A to a plot of FliM numbers in a motor-versus-cytoplasmic FliM concentration (Fig. S7A). The relatively wide range of observed FliM numbers in motor over the cell population may result from various experimental uncertainties, such as cell-to-cell variations in motor fluorescence intensity in a TIRF setup as the motor distance to the glass surface varies from cell to cell, possible occasional positioning of two motors within the diffraction limit, and uncertainties in counting the motor fluorescence intensity, etc.

From equation 2, *dN*/*dt* = 0 at steady state, thus

$$k_{\text{on}}U\left(M-N\right)-k_{\text{off}}(N-N_{\text{NE}})=0$$

For a CCW-rotating motor, *N*_NE_ ≈ 0.76 *N* according to a previous estimate, and *M* = 56 according to the estimate (12). Thus,

$$\frac{1}{N}=\frac{0.24}{56}\cdot \frac{k_{\text{off}}}{k_{\text{on}}}\cdot \frac{1}{U}+\frac{1}{56}$$

where *N* is the number of FliM molecules in a motor and *U* is the cytoplasmic FliM concentration. We converted Fig. S7A to a plot of 1/*N* versus 1/*U* and by fitting with the linear function obtained a value of *k*_off_/*k*_on_ of 914 ± 178 nM (Fig. S7B).
