# Supplementary figures and images for: Robustness in an Ultrasensitive Motor
Source: mBio. 2020 Mar 3;11(2):e03050-19. doi: 10.1128/mBio.03050-19 (PMC7064772; doi:10.1128/mBio.03050-19)

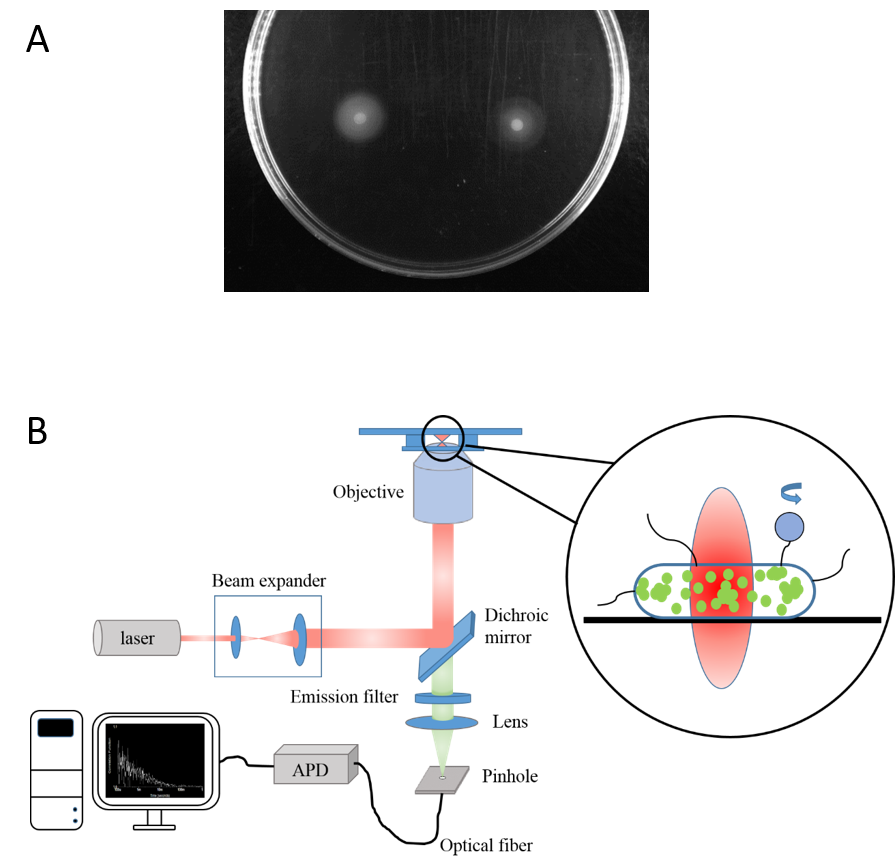

Supplement: FIG S1 [file mBio.03050-19-sf001.tif]

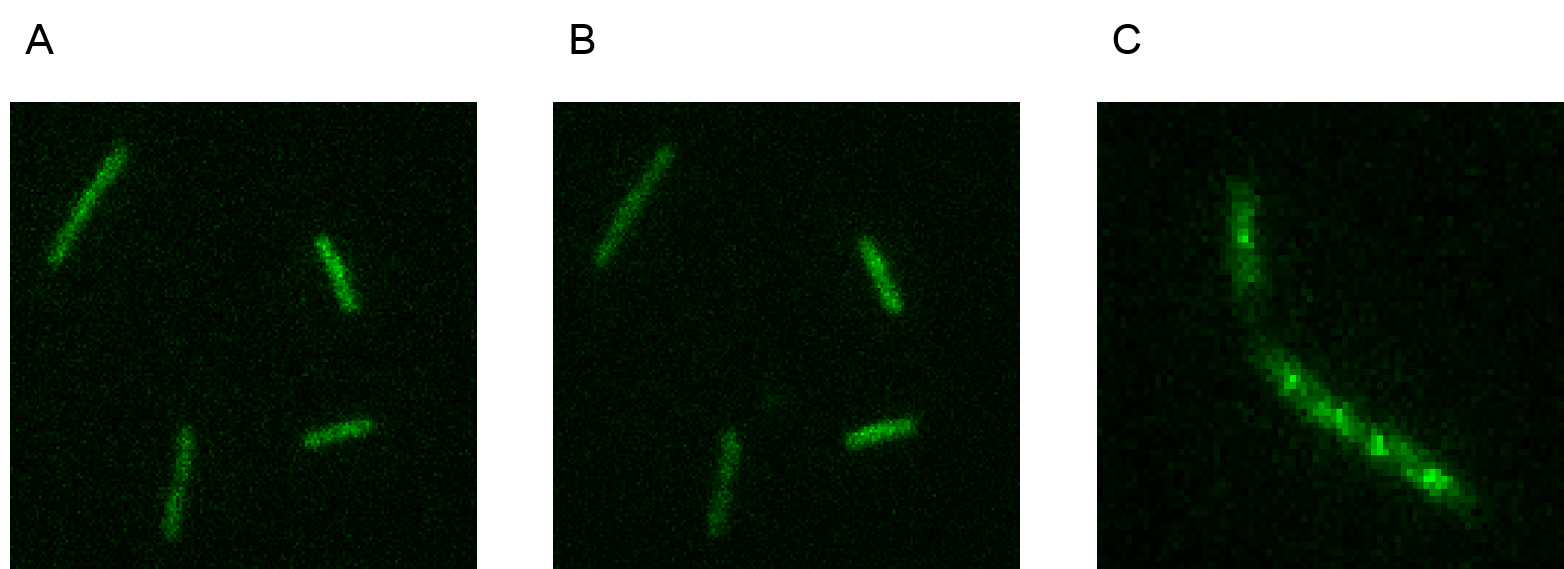

Supplement: FIG S5 [file mBio.03050-19-sf005.tif]
