# Supplementary material for: Robustness in an Ultrasensitive Motor
Source: mBio. 2020 Mar 3;11(2):e03050-19. doi: 10.1128/mBio.03050-19 (PMC7064772; doi:10.1128/mBio.03050-19)
Supplement: TEXT S1 [file mBio.03050-19-s0001.docx]

**Supplemental Methods**

**Model of robustness including the effect that FliM binding increases CheY-P lifetime.**

According to the previous study (28)，CheY-P that is bound to FliM is not susceptible to the action of the phosphatase CheZ. This means FliM in the cytoplasm will increase CheY-P lifetime. Therefore we considered this mechanism and incorporated it into our model of motor adaptive remodeling.

First, the chemotaxis signaling pathway of *E. coli* can be modeled by the following system of ordinary differential equations for the average cellular concentrations of each protein in the pathway (28):

$\left[ CheA \right]_{tot}=\left[ CheA \right]+\left[ CheA\text{-}P \right]$ (1)

$\left[ CheB \right]_{tot}=\left[ CheB \right]+\left[ CheB\text{-}P \right]$ (2)

$\left[ CheZ \right]_{tot}=\left[ CheZ \right]+\left[ CheZ\cdot CheY\text{-}P \right]$, (3)

$\left[ CheY \right]_{tot}=\left[ CheY \right]+\left[ CheY\text{-}P \right]+\left[ FliM\cdot CheY\text{-}P \right]+\left[ CheZ\cdot CheY\text{-}P \right]$ (4)

$\frac{d\left[ CheA\text{-}P \right]}{dt}=\alpha k_{cat}^{A}\left[ CheA \right]-\left[ CheA\text{-}P \right]\left( k_{a}^{Y}\left[ CheY \right]+k_{a}^{B}\left[ CheB \right] \right)$ (5)

$\frac{d\left[ CheB\text{-}P \right]}{dt}=k_{a}^{B}\left[ CheA\text{-}P \right]\left[ CheB \right]-k_{h}^{B}\left[ CheB\text{-}P \right]$ (6)

${\frac{d\left[ CheY\text{-}P \right]}{dt}=k}_{a}^{Y}\left[ CheA\text{-}P \right]\left[ CheY \right]-\left[ CheY\text{-}P \right]\left( k_{a}^{Z}\left[ CheZ \right]+k_{a}^{M}\left[ FliM \right]+k_{h}^{Y} \right)+ k_{d}^{Z}\left[ CheZ\cdot CheY\text{-}P \right]+k_{d}^{M}\left[ FliM\cdot CheY\text{-}P \right]$ (7)

$$\frac{d\left[ CheZ \right]}{dt}=k_{a}^{Z}\left[ CheZ \right]\left[ CheY\text{-}P \right]-\left( k_{d}^{Z}+k_{cat}^{Z} \right)\left[ CheZ\cdot CheY\text{-}P \right] \left( 8 \right)$$

$\frac{d\left[ FliM \right]}{dt}=\left( k_{d}^{M}+k_{h}^{Y} \right)\left[ FliM\cdot CheY-P \right]-k_{a}^{M}\left[ CheY-P \right]_{ub}\left[ FliM \right]$ (9)

Here, total concentrations are denoted by the subscript tot, phosphorylated species are denoted by –P, and complexes are denoted by a dot between the two species names (e.g., CheZ·CheY-P). As we focused on the adapted steady states, the rates of change in equations 5−9 were set to zero, and the active fraction α of CheA was modeled as

$\alpha=\frac{k_{cat}^{R}\left[ CheR \right]_{tot}}{\left( k_{cat}^{R}\left[ CheR \right]_{tot}+k_{cat}^{B}\left[ CheB\text{-}P \right] \right)}$ (10)

Binding of CheY-P to FliM was modeled using the Hill function:

$\left[ FliM\cdot CheY\text{-}P \right]=\left[ FliM \right]_{tot}\times\frac{\left[ CheY\text{-}P \right]^{n}}{\left( \left[ CheY\text{-}P \right]^{n}+K_{d}^{n} \right)}$ (11)

with *n* = 1.7 and *K*_d_ = 3100 nM. All other parameters (total concentrations except [FliM]_tot_, and reaction rates) were taken from ref. 28. The number of FliM molecules *N* in a motor and CW bias *B* were related by the following two equations as described before:

$$k_{on}U\times\left( M-N \right)-k_{off}\left( N-\left( B\times12+\left( 1-B \right)\times34 \right) \right)=0 (12)$$

$$B=1/(1+exp(N\times\ln\left( \frac{1+\frac{\left[ CheY\text{-}P \right]}{K_{2}}}{1+\frac{\left[ CheY\text{-}P \right]}{K_{1}}} \right)+\varepsilon)) (13)$$

At a specific [FliM]_tot_, the concentration of unbound CheY-P ([CheY-P]) can be extracted by solving equations 1−11 at steady state. Then the steady-state CW bias (*B*) can be extracted by solving the combination of equations 12 and 13 if a value of *ε* was assumed. To obtain a steady-state CW bias of 0.3 under our experimental condition, we calculated the total concentration of FliM by using the linear dependence between [FliM]_tot_ and cytoplasmic FliM (*U*) (see Fig. S4). The value of *ε* was determined to be 24.4 *k*_B_T according to the estimation of 600 nM cytoplasmic FliM concentration in wildtype cells.

We then calculated the dependence of motor CW bias on the cytoplasmic FliM concentration *U*. At each value of the cytoplasmic FliM concentration, the [FliM]_tot_ was obtained from the linear dependence in Fig. S4, and the concentration of unbound CheY-P was calculated by solving the combination of equations 1 − 11. To calculate the dependence with the effect of CheY-P binding to FliM and that this binding increases CheY-P lifetime (green dashed line in Fig. S9), the motor response curve with Hill coefficient of 20.7 was used to obtain the motor CW bias ([9](#_ENREF_9)). To combine these effects with the effect of motor adaptive remodeling (red solid line in Fig. S9), the combination of equations 12 and 13 was solved to obtain the motor CW bias.
